# TRPV1 function is modulated by Cdk5-mediated phosphorylation: insights into the molecular mechanism of nociception

**DOI:** 10.1038/srep22007

**Published:** 2016-02-23

**Authors:** Thomas Jendryke, Michaela Prochazkova, Bradford E. Hall, Grégory C. Nordmann, Moritz Schladt, Vladimir M. Milenkovic, Ashok B. Kulkarni, Christian H. Wetzel

**Affiliations:** 1 Molecular Neurosciences, Department of Psychiatry and Psychotherapy, University of Regensburg, 93053 Regensburg, Germany; 2 Functional Genomics Section, Laboratory of Cell and Developmental Biology, National Institute of Dental and Craniofacial Research, National Institutes of Health, Bethesda, MD 20892, USA

## Abstract

TRPV1 is a polymodally activated cation channel acting as key receptor in nociceptive neurons. Its function is strongly affected by kinase-mediated phosphorylation leading to hyperalgesia and allodynia. We present behavioral and molecular data indicating that TRPV1 is strongly modulated by Cdk5-mediated phosphorylation at position threonine-407(mouse)/T406(rat). Increasing or decreasing Cdk5 activity in genetically engineered mice has severe consequences on TRPV1-mediated pain perception leading to altered capsaicin consumption and sensitivity to heat. To understand the molecular and structural/functional consequences of TRPV1 phosphorylation, we generated various rTRPV1_T406_ receptor variants to mimic phosphorylated or dephosphorylated receptor protein. We performed detailed functional characterization by means of electrophysiological whole-cell and single-channel recordings as well as Ca^2+^-imaging and challenged recombinant rTRPV1 receptors with capsaicin, low pH, or heat. We found that position T406 is critical for the function of TRPV1 by modulating ligand-sensitivity, activation, and desensitization kinetics as well as voltage-dependence. Based on high resolution structures of TRPV1, we discuss T406 being involved in the molecular transition pathway, its phosphorylation leading to a conformational change and influencing the gating of the receptor. Cdk5-mediated phosphorylation of T406 can be regarded as an important molecular switch modulating TRPV1-related behavior and pain sensitivity.

The *Transient receptor potential vanilloid 1* (TRPV1) is a ligand-gated non-selective cation channel which is prominently expressed in sensory nociceptive C- and Aδ fibers of trigeminal and dorsal root ganglia neurons^1^,^2^. Common for all TRP channels is a tetrameric structure, with each subunit including six transmembrane domains (TMD1-TMD6) and extensive intracellular amino and carboxyl termini^3^. As a polymodal receptor, TRPV1 is activated by various exogenous and endogenous stimuli such as the vanilloid capsaicin, heat^2^, and protons^4^, as well as the endocannabinoid anandamide^5^. During inflammation, sensory neurons are sensitized by inflammatory mediators, which activate several signal transduction pathways, leading to protein kinase-mediated phosphorylation of TRPV1^6^,^7^. Several protein kinases such as PKA^8^,^9^,^10^, PKC^11^,^12^,^13^, CaMKII^14^, and c-Src kinase^15^, are known to phosphorylate TRPV1 at various serine and threonine residues, leading to sensitization of receptor function. In contrast, dephosphorylation of TRPV1 by the Ca^2+^-dependent phosphatase calcineurin leads to the desensitization of the receptor^16^.

In 2007, Pareek *et al.* reported that cyclin-dependent kinase 5 (Cdk5)-mediated phosphorylation of TRPV1 regulates Ca^2+^ influx through this channel. They demonstrated that Cdk5 deficiency in sensory neurons of mice abrogated TRPV1 phosphorylation and induced thermal hypoalgesia. Analyzing the amino acid sequence of TRPV1 revealed three different potential consensus sites for Cdk5-mediated phosphorylation: threonine-108 (T108), threonine-407 (T407), and serine-612 (S612). Of the three sites, T407 is highly conserved and the preferred target site for Cdk5-dependent phosphorylation^17^. In order to further investigate pain signal transduction in nociceptive neurons, we functionally characterized the impact of Cdk5-mediated phosphorylation on the TRPV1 receptor. We studied TRPV1-mediated pain responses in genetically engineered mice with either increased or decreased Cdk5 activity. Mouse behavioral testing included quantification of *in vivo* sensitivity to oral capsaicin as well as measuring the sensitivity to facial contact with thermodes set at 45 °C, a noxious temperature known to activate TRPV1. Additionally, we set out to functionally and biophysically characterize recombinant wild-type or mutant rat TRPV1 receptors heterologously expressed in HEK293T and CHO cells. We conducted both whole-cell and single-channel patch-clamp recordings, as well as Ca^2+^ imaging. Site-directed mutagenesis was used to replace T406 in the rat TRPV1 (corresponding to T407 in mice and humans) by alanine (T406A) or by aspartate (T406D), in order to block Cdk5-mediated phosphorylation at this position, or to mimic the effect of phosphorylation by introducing a bulky and negatively charged residue, respectively. In our behavioral studies, we found that mice with reduced Cdk5 activity showed higher tolerance to TRPV1-mediated painful stimuli compared to wild-type mice, whereas mice with an increased Cdk5 activity were significantly less tolerant to the same stimuli. Our functional analysis at the molecular and cellular level revealed that modification of the T406 residue in the rat TRPV1 dramatically affects important functional parameters such as receptor sensitivity, voltage-dependence, and kinetics of activation and desensitization. These changes in TRPV1 function due to T406 mutation suggest that T406 phosphorylation by Cdk5 can be regarded as the molecular basis for the altered behavior and pain sensitivity seen in our genetically engineered mouse model. Moreover, with regard to the high resolution structures of the rat TRPV1 which was solved by Liao^18^ and Cao^19^ in 2013, we discuss the impact of the conformational changes induced by phosphorylation of T406 on the 3D structure of the receptor protein. The proposed conformational changes may (at least in part) explain the functional changes we observed at both the molecular and behavioral level.

## Results

First, we wanted to determine the impact of Cdk5 on TRPV1 activity in response to stimuli that are known to activate TRPV1 *in vivo.* Therefore, we examined the sensitivity of genetically engineered mice that either have increased Cdk5 activity (by overexpression p35, Tgp35) or decreased Cdk5 activity (either by knocking out p35 or through conditional deletion of Cdk5 in nociceptive neurons, Cdk5CoKo).

### Cdk5 activity regulates aversion to oral capsaicin in mice

**Figure 1:**
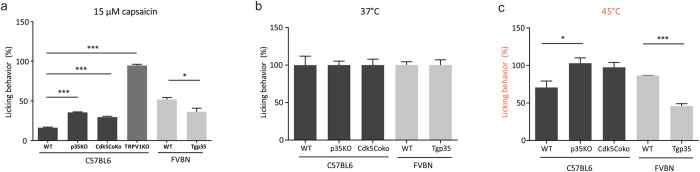
Responses of wild-type, p35KO, Cdk5CoKo, TRPV1KO, and Tgp35 mice to capsaicin and heat. Water-deprived C57Bl6 and FVBN mice were tested for 1 h using the lickometer with a free access to water containing 15 μM capsaicin. The behavior is expressed as a % of the baseline licking responses for plain water as compared to capsaicin. Increased aversion and hypersensitivity to capsaicin was evident in Tgp35 mice (FVBN background) by decreased number of licks (unpaired t-test, p < 0.05). In contrast, p35 knockout or Cdk5CoKo mice (C57BL6/129SVJ background) showed less aversion to capsaicin compared to their littermate wild-type (WT) controls (One-way ANOVA followed by Dunnett’s multiple comparisons test, p < 0.0001). Data are presented as mean ± SEM from four animals during five different measurements (**a**). Effect of temperature activation of TRPV1 in mutant animals. An orofacial pain assessment device was used to measure the responses of the mice to hot facial stimulation. All mice showed similar consumption of the reward (sucrose) at 37 °C (**b**). Tgp35 mice displayed an aversive behavior to the increased temperature of the thermodes as noted by significantly decreased licking behavior (unpaired t-test, p = 0.0002), whereas p35KO mice displayed significantly increased number of licks compared to wild-type controls (One-way ANOVA followed by Dunnett’s multiple comparisons test, p < 0.05) (**c**). Data are presented as mean ± SEM from four animals measured five times using 37 °C and three times at 45 °C.

We have previously reported that Cdk5 is able to phosphorylate TRPV1, which, in turn, influences thermal nociception. Conditional deletion of Cdk5 in nociceptive neurons abrogates phosphorylation of TRPV1, which possibly contributes to the thermal hypoalgesia seen in these mice^17^. To further examine the interaction between Cdk5 activity and TRPV1 function, we tested the sensitivity of our mice to capsaicin (15 μM), a specific activator of TRPV1. Oral administration of capsaicin causes an unpleasant burning sensation, so we used the lickometer to measure aversion to water containing this TRPV1 agonist. During the training sessions (water only), there was no change in the licking behavior between the different genotypes of mice and their corresponding controls. After habituation to the lickometer, capsaicin was added to the drinking water. In mice with increased Cdk5 activity, we saw increased aversion to 15 μM capsaicin as evident by the decreased number of licks (unpaired t-test p < 0.05). In contrast, p35KO as well as Cdk5 CoKo mice showed decreased aversion to capsaicin consumption (One-way ANOVA followed by Dunnett’s multiple comparison test, p < 0.0001) (Fig. 1a). We have validated these results using TRPV1KO mice as a positive control, which show no difference in their capsaicin consumption. These results confirm that Cdk5 activity modulates oral pain related responses transduced via the TRPV1 channel.

### Cdk5 activity modulates thermal nociceptive signaling in orofacial area

Since TRPV1 is a poly-modal ion channel, we additionally wanted to examine thermal nociception at a temperature known to activate TRPV1. To measure orofacial thermosensitivity, we used the OPAD system (Orofacial Pain Assessment Device, Stoelting), an operant behavioral testing device that provides an automated measurement of both hot and cold-induced noxious orofacial stimuli. We observed that both p35KO and transgenic p35 mice exhibit altered responses to thermal stimulation. We did not observe any difference in 30% sucrose consumption at 37 °C between the genotypes (Fig. 1b). However, we observed increased aversion to facial contact with thermodes set at 45 °C in mice overexpressing p35, as was evidenced by the decreasing number of attempts the mice made to access the reward (unpaired t-test, p = 0.0002, Fig. 1c). The number of reward licking/facial contact events was also significantly decreased in these mice when the test temperature was increased. To the contrary, mice with decreased Cdk5 activity displayed thermal hypoalgesia (One-way ANOVA followed by Dunnett’s multiple comparison test, p < 0.05, Fig. 1c). Using the lickometer and OPAD behavioral devices, we were able to test two known activators of TRPV1, capsaicin and noxious heat in our genetically engineered mouse models with altered Cdk5 activity. Our behavioral studies indicate that Cdk5 activity modulates TRPV1 channel activity *in vivo*, and that increased TRPV1 sensitivity is probably a result of direct phosphorylation of TRPV1 by Cdk5.

### Ca^2+^-induced desensitization of TRPV1 is modulated by co-expression of Cdk5 and p35

**Figure 2:**
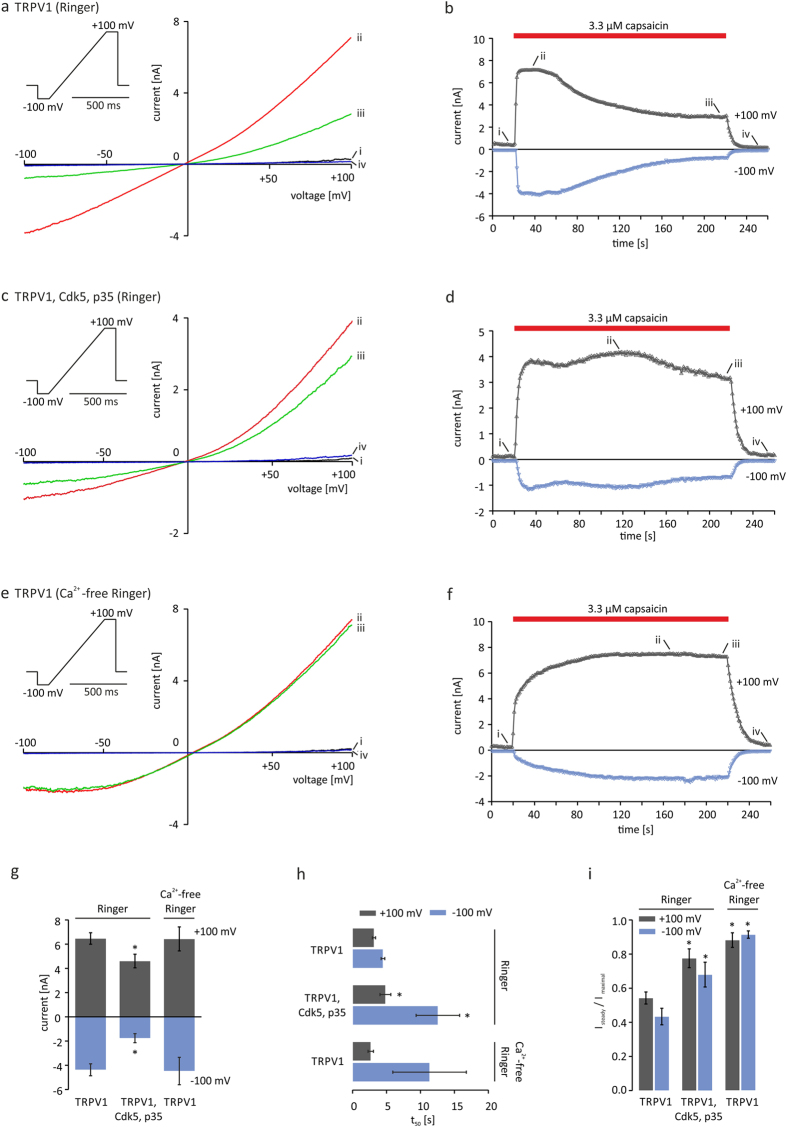
Cdk5-mediated phosphorylation of TRPV1 prevents desensitization to capsaicin. TRPV1-mediated inward (−100 mV) and outward (+100 mV) currents in transiently transfected CHO cells induced by 3.3 μM capsaicin in the presence or absence of extracellular Ca^2+^. Left panels show I/V relationships corresponding to the representative recording traces on the right. In the presence of extracellular Ca^2+^, application of capsaicin for 200 s established a desensitized steady state in TRPV1 expressing cells (**a,b**). Co-expression of TRPV1, Cdk5-mCherry and p35-CFP inhibits the Ca^2+^-induced desensitization (**c,d**), similar to the capsaicin-induced currents of TRPV1 in absence of extracellular Ca^2+^ (**e,f**). Maximal induced currents (**g**), time to half-maximal response represented as t_50_ (**h**) and desensitization as ratio I_steady_/I_maximal_ (**i**) of n = 11–22 independent measurements. Asterisk (*) indicates significant differences compared to the corresponding TRPV1 value at Ca^2+^-containing conditions (unpaired *WR-*test, p < 0.05).

Next, we wanted to determine in more detail how Cdk5-mediated phosphorylation affects TRPV1 channel function by using the patch-clamp technique. To this end, we tested TRPV1 activity in CHO cells co-expressing rTRPV1 and GFP, or co-expressing a combination of rTRPV1, Cdk5-mCherry, and p35-CFP. The fluorescent tags were used to visually select CHO cells expressing the respective proteins for whole-cell voltage-clamp recordings. Voltage-ramp protocols (−100 mV to +100 mV) were applied in order to analyze inward and outward currents. To induce TRPV1-mediated currents, 3.3 μM capsaicin was applied for 200 s in Ca^2+^-containing Ringer’s solution (solution A). Capsaicin induced fast activating TRPV1-mediated currents characterized by a strong acute desensitization in both inward and outward direction (Fig. 2a,b). The desensitization of currents could be prevented by removing extracellular Ca^2+^ (solution B) (Fig. 2e,f). Interestingly, 3.3 μM capsaicin also induced non-desensitizing currents even in presence of Ca^2+^, with slightly reduced activation kinetics, after the co-expression of TRPV1, Cdk5 and p35 (Fig. 2c,d), suggesting that Cdk5-mediated phosphorylation of TRPV1 is responsible for changing TRPV1 activity into a non-desensitizing state. Moreover, we investigated the effect of Cdk5-mediated phosphorylation on the TRPV1 capsaicin concentration-response relationship under Ca^2+^-free non-desensitizing conditions. Therefore, CHO cells expressing TRPV1 or co-expressing TRPV1, Cdk5 and p35 were recorded in the whole-cell configuration and voltage-ramp protocols were used to characterize TRPV1-mediated currents evoked by various capsaicin concentrations. Supplemental Fig. 1 shows representative recordings of capsaicin-induced currents of CHO cells expressing TRPV1 or TRPV1, Cdk5 and p35. The TRPV1-mediated currents were normalized by referring the respective current amplitudes to the current evoked by application of 3.3 μM capsaicin in the same cell, and the Hill-equation (Equation 1) was used to calculate the EC_50_. For TRPV1 measurements, EC_50_ values were calculated to 0.25 ± 0.05 μM (out) and 0.63 ± 0.13 μM (in), whereas co-expression of TRPV1, Cdk5 and p35 resulted in EC_50_ values of 0.28 ± 0.04 μM (out) and 0.55 ± 0.08 μM (in). The statistical analysis revealed no significant Cdk5-mediated sensitization of capsaicin-induced TRPV1-mediated currents. As previously shown, the Cdk5-mediated phosphorylation of TRPV1 has severe physiological consequences on the perception and transduction of noxious stimuli. Therefore, we hypothesize that the reduced Ca^2+^-dependent desensitization found in cells co-expressing TRPV1, Cdk5 and p35 leads to increased receptor efficacy and promotes the development of allodynia and hyperalgesia in sensory neurons.

### TRPV1_T406_ mutagenesis affects the Ca^2+^-induced desensitization

**Figure 3:**
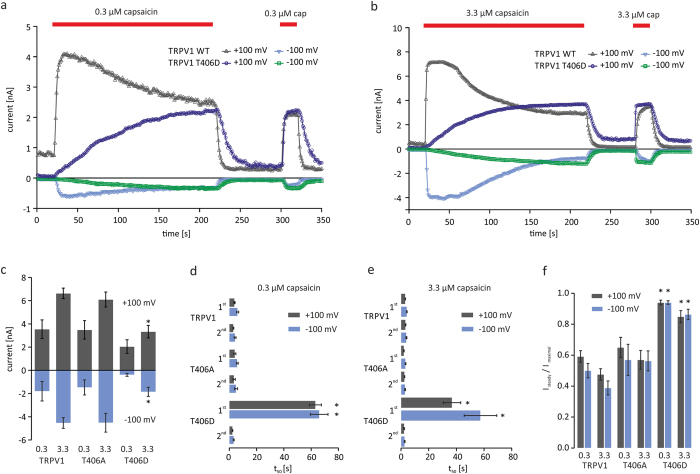
TRPV1_T406D_ mutants show slowed activation kinetics. Ca^2+^-induced desensitization of TRPV1_WT_ and TRPV1_T406_ mutants in transiently transfected CHO cells challenged with 0.3 μM or 3.3 μM capsaicin measured by voltage ramp protocols in the presence of extracellular Ca^2+^. Application of 0.3 μM (**a**) or 3.3 μM (**b**) capsaicin for 200 s induces TRPV1-mediated currents. Amplitudes of capsaicin-induced currents (**c**), activation kinetics represented by time to half maximum current (t_50_) of first and second response to 0.3 μM (**d**) or 3.3 μM capsaicin (**e**). Desensitization represented as ratio (I_steady_/I_maximal_) of n = 8–13 independent measurements (**f**). Asterisk (*) indicates significant differences compared to the corresponding TRPV1_WT_ value (unpaired *WR-*test, p < 0.05).

Pareek *et al.* demonstrated that the conditional deletion of Cdk5 in pain-sensing neurons in mice abrogates TRPV1 phosphorylation at T407^17^ and our results demonstrate that the co-expression of TRPV1, Cdk5 and p35 leads to the reduction of Ca^2+^-dependent desensitization. Based on these observations, we set out to further characterize the functional consequences of steric and electrical alterations at this particular position on functional parameters of the TRPV1 protein. Therefore, the corresponding threonine of the rat TRPV1 (T406) was replaced by different amino acids comprising different polar, nonpolar, aromatic, or charged characteristics. Transfected CHO cells were recorded in the presence of 2 mM Ca^2+^ (solution A) and TRPV1-mediated currents were induced by applying 3.3 μM capsaicin for 200 s. Mutations that alter steric and electrical properties at position 406 differentially affected the TRPV1 ion channel properties such as kinetics, amplitude and desensitization. Since the size of the recorded cells was consistent at 24.9 ± 2.4 pF (n = 91), we consider that the observed variability of the induced TRPV1-mediated currents were due to different expression rates (Supplemental Fig. 2a). The activation kinetics of mutant receptors carrying aspartate-406, or glutamate-406, as well as lysine-406 or proline-406 were found to be reduced (Supplemental Fig. 2b). Moreover, the desensitization was reduced or even eliminated by the exchange of T406 to negatively charged amino acids, as well as lysine, histidine and proline (Supplemental Fig. 2c). Due to its structure, aspartic acid is the most appropriate amino acid to mimic phosphorylation of proteins^20^. Currents of TRPV1 receptor variants were induced by 0.3 μM (Fig. 3a) and 3.3 μM capsaicin (Fig. 3b) in the presence of extracellular Ca^2+^. In contrast to TRPV1_WT_ and TRPV1_T406A_, the latter designed to inhibit endogenous Cdk5-mediated phosphorylation, the TRPV1_T406D_ mutant showed considerably modified activation kinetics. A first application of 0.3 μM or 3.3 μM capsaicin induced currents showing markedly decelerated activation kinetics, indicated by the increased time to half maximal amplitude (t_50_) when compared to TRPV1_WT_. The t_50_ of outward currents induced by 0.3 μM capsaicin was on average 3.9 ± 0.4 s in TRPV1_WT_ and 63.1 ± 4.4 s in TRPV1_T406D_ (p < 0.05). Interestingly, a second application of the identical stimulus after priming the cells with a high capsaicin concentration induced a fast activating response of TRPV1_T406D_ with t_50_ values similar to TRPV1_WT_ and TRPV1_T406A_ (TRPV1_WT_: 3.0 ± 0.3 s; TRPV1_T406A_: 3.5 ± 0.9 s TRPV1_T406D_: 2.4 ± 0.2 s) (Fig. 3d,e) suggesting a use-dependent behavior of the TRPV1_T406D_ receptor mutant. Similar to the phosphorylated TRPV1 (after co-expression of TRPV1, Cdk5, and p35), TRPV1_T406D_ did not show any desensitization. The desensitization of inward currents (measured as I_steady_/I_maximal_) induced by 0.3 μM capsaicin was on average 0.50 ± 0.05 in TRPV1_WT_ and 0.94 ± 0.01 in TRPV1_T406D_ (Fig. 3f). Interestingly, in TRPV1-mediated responses of cells co-expressing TRPV1_T406D_, Cdk5, and p35, we found no difference in activation and desensitization kinetics compared to cells only express TRPV1_T406D_ (Supplemental Fig. 3). Next we set out to investigate the use-dependent behavior of TRPV1_T406D_ in detail. In order to analyze the molecular mechanisms that lead to the altered activation kinetics of TRPV1_T406D_, and to address the question whether changes in response kinetics might be dependent on dynamic receptor trafficking, we monitored the dynamics of membrane localization of directly C-terminally GFP-tagged TRPV1_T406_ receptor variants. Electrophysiological recordings of GFP-TRPV1_T406,_ GFP-TRPV1_T406D_, and GFP-TRPV1_T406A_ receptor variants demonstrated that they were fully functional and showed the characteristic functional behavior of the respective untagged receptors (Supplemental Fig. 4). Using TIRF microscopy to visualize membrane expression of the fluorescent TRPV1 receptors, we could not detect any change in fluorescence of GFP-TRPV1_T406_ receptors in the membrane during or after application of a supra-maximum concentration of capsaicin (6.6 μM) (Supplemental Fig. 5), indicating that the density/expression of TRPV1 receptors in the membrane is not altered during capsaicin treatment. Interestingly, in addition to the markedly altered channel activation and desensitization kinetics, we observed that the TRPV1_T406D_-mediated outward currents (at +100 mV) after maximal stimulation did not reach baseline, but stayed at a higher activation level (Fig. 3b), which is suggestive of an altered voltage-dependence.

### Targeted mutagenesis affects the voltage-dependence of TRPV1

**Figure 4:**
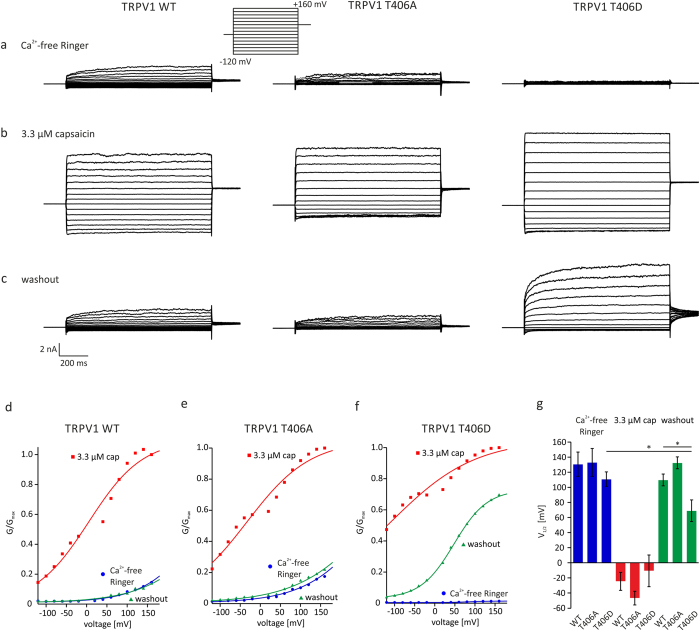
Voltage-dependence of TRPV1 is altered in the T406D mutant. Voltage-dependence of TRPV1_WT_ and TRPV1_T406_ mutants in transiently transfected CHO cells measured by voltage step protocols with depolarizing pulses from −120 mV to +160 mV. In Ca^2+^-free ringer solution, voltage-dependent currents are detected in TRPV1_WT_ and TRPV1_T406A_, but not in TRPV1_T406D_ (**a**). Application of 3.3 μM capsaicin induces robust voltage-dependent currents in CHO cells expressing TRPV1_WT_, TRPV1_T406A_ or TRPV1_T406D_ (**b**). Voltage-activated currents evoked one minute after washout of capsaicin revealed an increased voltage-dependence of TRPV1_T406D_, whereas the voltage-induced currents of TRPV1_WT,_ TRPV1_T406A_ recover to the same level as under Ringer’s conditions (**c**). Normalized conductance G/G_max_ of n = 6–7 independent measurements of TRPV1_WT_ (**d**), TRPV1_T406A_ (**e**), and TRPV1_T406D_ (**f**). Sigmoidal fit was used to calculate V_1/2_. Asterisk (*) indicates significant decrease of V_1/2_ of TRPV1_T406D_ after priming with 3.3 μM capsaicin (paired *WR-*test, p < 0.05).

To investigate the voltage-dependence of TRPV1_T406D_, voltage-induced currents were measured before, during, and after 3.3 μM capsaicin stimulation under Ca^2+^-free conditions (solution B) by applying defined voltage-steps (−120 mV to +160 mV). TRPV1_WT_ and TRPV1_T406A_ exhibited the characteristic outward rectifying voltage-activated currents, whereas hardly any currents could be recorded in TRPV1_T406D_ mutants (Fig. 4a). However, application of 3.3 μM capsaicin resulted in robust inward and outward currents in TRPV1_WT_, TRPV1_T406A_, and TRPV1_T406D_ (Fig. 4b) with a maximal current of about 0.6 pA/pF at +160 mV. Finally, voltage-activated currents were recorded 1 min after the washout of capsaicin. For detailed analysis of the voltage-dependence of TRPV1_WT_, TRPV1_T406A_, and TRPV1_T406D_, the conductance G was normalized by calculating the G/G_max_ ratio, and plotted against the applied voltage. Since the conductance/voltage-relationship is a direct measure of the voltage-dependence, we analyzed this parameter by mathematically approximating the data using a sigmoidal fit (Equation 2) in order to estimate V_1/2_ (see Methods). We found a considerably increased conductance and left shift of the curve paralleled by a significant reduction in V_1/2_ for TRPV1_T406D_ from +110 to +69 mV after priming with 3.3 μM capsaicin (paired *WR*-test, p < 0.05) (Fig. 4d–g). Furthermore, we measured the voltage-dependence of TRPV1_T406D_ 2, 3, and 5 min after the capsaicin washout and found that the altered voltage-dependence of TRPV1_T406D_ persisted even 5 min after the capsaicin-induced activation (Supplemental Fig. 6). Our data clearly demonstrate that the mutation of T406 to the negatively charged aspartic acid (T406D) strongly affects the voltage-dependence of TRPV1 receptors.

### TRPV1_T406_ mutations modify the sensitivity to capsaicin

**Figure 5:**
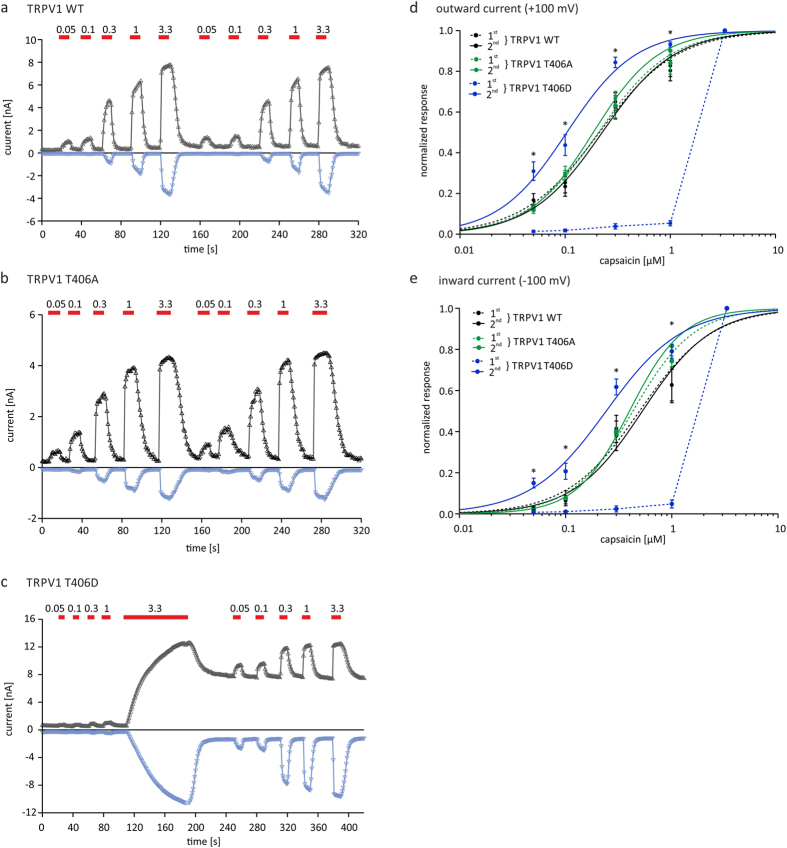
Sensitivity of TRPV1 to capsaicin is altered in the T406D mutant. Analysis of TRPV1 concentration/response-relationships in transiently transfected CHO cells using voltage-ramp protocols in the absence of extracellular Ca^2+^. Inward and outward currents of TRPV1_WT_ (**a**), TRPV1_T406A_ (**b**) and TRPV1_T406D_ (**c**) induced by 0.05, 0.1, 0.3, 1 and 3.3 μM capsaicin. Concentration/response-relationships of outward (**d**) or inward (**e**) currents of TRPV1_WT_ and TRPV1_T406A_ show no difference between first and second series of application. Before priming of TRPV1_T406D_ with 3.3 μM capsaicin, responses were small and not evaluable. Challenging cells with capsaicin (3.3 μM) led to significant increase in sensitivity and allowed the TRPV1_T406D_ receptor to then respond to lower concentrations (paired *t-*test, p < 0.05). EC_50_ values obtained during the second series of capsaicin application (outward: TRPV1_WT_ 0.28 ± 0.06 μM; TRPV1_T406A_ 0.19 ± 0.02 μM; TRPV1_T406D_ 0.11 ± 0.01 μM; inward: TRPV1_WT_ 0.66 ± 0.14 μM; TRPV1_T406A_ 0.40 ± 0.07 μM; TRPV1_T406D_ 0.26 ± 0.03 μM). n = 6–10 independent measurements were performed for each receptor variant.

In order to investigate the effect of T406 mutation on receptor sensitivity to capsaicin, we analyzed the concentration/response-relationship of TRPV1_WT_ and TRPV1_T406_ mutants by applying various capsaicin concentrations (0.05 to 3.3 μM). Currents were recorded under Ca^2+^-free conditions to prevent desensitization of the receptors during repetitive agonist applications. Due to the use-dependent activation pattern observed for TRPV1_T406D_, we analyzed the capsaicin concentration/response-relationship in two consecutive sets of application. Supplemental Table 1 presents the EC_50_ values revealed for inward and outward currents. While TRPV1_WT_ and TRPV1_T406A_ showed no difference in apparent affinity between the first and second set (Fig. 5a,b), TRPV1_T406D_ showed a marked increase in sensitivity. The first set of applications did not evoke currents in response to low concentrations of capsaicin, while robust currents developed during application of the maximum stimulus (3.3 μM capsaicin). The following set of applications induced markedly increased currents already at low capsaicin concentrations (Fig. 5c). Because of the hardly detectable current responses to low capsaicin concentrations during the first set, the accurate estimation of the EC_50_ is not applicable. However, the second set of applications induced currents that correlated with capsaicin concentration and allowed the calculation of EC_50_ values for TRPV1_T406D_. At +100 mV, the EC_50_ was significantly reduced in TRPV1_T406D_ compared to TRPV1_WT_ (0.11 ± 0.01 μM vs. 0.28 ± 0.06 μM; p < 0.05) (Fig. 5d,e). Based on these findings, we conclude that electrical and steric properties of the amino acid residue at position 406 strongly influence the capsaicin sensitivity of TRPV1.

### TRPV1_T406_ mutagenesis also affects proton and heat activation

**Figure 6:**
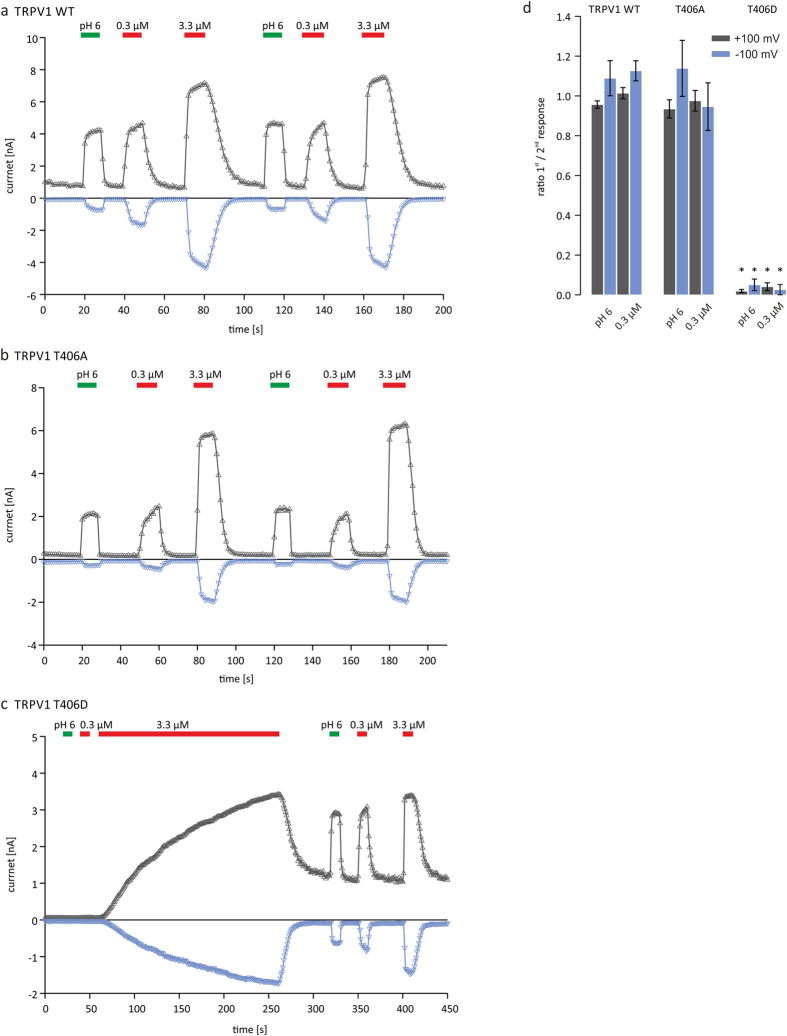
Sensitivity of TRPV1 to pH 6 is altered in the T406D mutant. pH 6, 0.3 μM and 3.3 μM capsaicin-induced currents of TRPV1_WT_ and TRPV1_T406_ mutants in the absence of extracellular Ca^2+^. Application of Ringer’s solution at pH 6 or 0.3 μM capsaicin induces currents in TRPV1_WT_ (**a**) and TRPV1_T406A_ (**b**) receptor variants. TRPV1_T406D_ expressing cells respond to pH 6 or 0.3 μM capsaicin only after priming the cells with 3.3 μM capsaicin (**c**). Ratio between first and second response indicates sensitization (<1) or desensitization (>1) during 3.3 μM capsaicin priming (**d**). Analysis of n = 10–15 independent measurements shows that TRPV1_T406D_ is sensitized by priming with 3.3 μM capsaicin (unpaired *WR-*test, p < 0.05).

**Figure 7:**
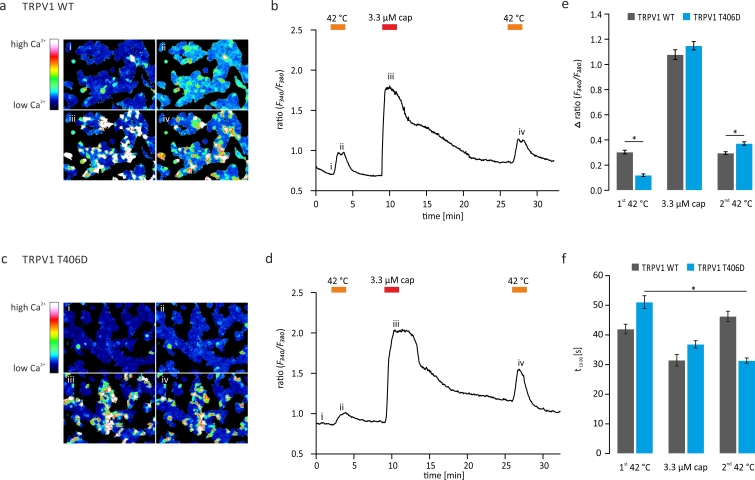
Response of TRPV1 to heat is altered in the T406D mutant. HEK293T cells expressing TRPV1_WT_ or TRPV1_T406D_ in Fura-2 Ca^2+^ imaging experiments. Representative ratio image and time course of TRPV1 n = 131 (**a,b**) and TRPV1_T406D_ n = 136 (**c,d**) measurements. Mean ± SEM of the TRPV1-mediated responses (Δ ratio (*F*_340_/*F*_380_)), induced by 42 °C or 3.3 μM capsaicin (**e**). Responses to first and second heat stimuli were equal in TRPV1_WT_ expressing cells. Compared to TRPV1_WT_, the initial response of TRPV1_T406D_ to heat (42 °C) is significantly lower, but increases after priming with 3.3 μM capsaicin (paired *t*-test, p < 0.05). The activation kinetics (rise time from 10 to 90% = t_10–90_) of TRPV1_T406D_ is significantly accelerated in the second heat-induced responses (1^st^ 51.1 ± 2.2 s, 2^nd^ 31.3 ± 0.8 s) (paired *t*-test, p < 0.05) (**f**).

Since the TRPV1 receptor is activated by various noxious stimuli, such as voltage, capsaicin, heat, or protons, we set out to study the dependence of TRPV1_WT_, TRPV1_T406A_, and TRPV1_T406D_ on the modality of the activating stimulus. To this end, cells expressing the respective receptor variants were challenged with two consecutive sets of applications of pH 6, as well as 0.3 μM and 3.3 μM capsaicin under Ca^2+^-free conditions (Fig. 6a–c). While TRPV1_WT_ and TRPV1_T406A_ responded to pH 6, 0.3 μM, and 3.3 μM capsaicin during the first and second set of application, TRPV1_T406D_ responded during the first set only to 3.3 μM capsaicin, but gained full responsiveness to low pH and capsaicin during the second set, again reflecting a use-dependent activation pattern. The ratio of the first and second response to the same stimulus is a measure of sensitization or desensitization of the receptor (Fig. 6d and Supplemental Fig. 2d). In order to investigate the sensitivity of TRPV1 receptor mutants to heat, we performed Fura-2 Ca^2+^ imaging of HEK293T cells expressing TRPV1_WT_ or TRPV1_T406D_ by making use of the high Ca^2+^ permeability of the TRPV1 ion channel. We evoked TRPV1-mediated Ca^2+^ fluxes by stimulating the cells with 3.3 μM capsaicin at room temperature, or by perfusing the recording chamber with physiological Ringer’s solution (solution A) heated up to >42 °C. Figure 7 represents characteristic Ca^2+^ imaging measurements of HEK293T cells expressing the TRPV1_WT_ or TRPV1_T406D_, respectively. Cells were challenged by heating the bath solution to 42 °C followed by application of 3.3 μM capsaicin at room temperature (24 °C). The second heat activation was performed 15 min after the washout of 3.3 μM capsaicin (Fig. 7a–d). The functional properties of TRPV1 were analyzed by evaluating the Δratio (*F*_340_/*F*_380_), as a measure for the heat or capsaicin-induced TRPV1-mediated Ca^2+^ influx. In TRPV1_WT_ measurements, no difference between the first and second Ca^2+^ response to heat was detected, whereas in recordings of TRPV1_T406D_ expressing cells, a decreased response to the first and an increased response to the second heat stimulus was observed (Fig. 7e). The increased efficacy of TRPV1_T406D_ was paralleled by an acceleration in activation kinetics (Fig. 7f). Our Ca^2+^ imaging results are in line with our previous electrophysiological data on TRPV1_T406D_ function and suggest that mutation of T406 affects the polymodal activation properties of TRPV1, probably by altering the voltage-dependence of the receptor.

### Single-channel characteristics of TRPV1_T406D_ are different from those of TRPV1_WT_

**Figure 8:**
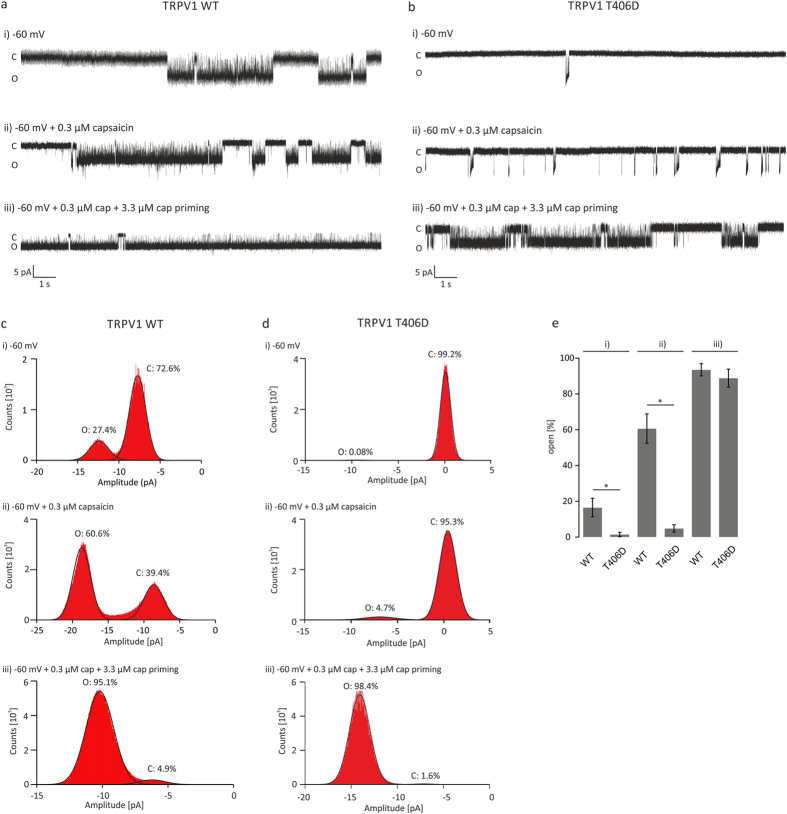
TRPV1 single-channel properties are altered in the T406D mutant. TRPV1 single-channel events measured in cell-attached configuration of transiently transfected CHO cells at −60 mV pipette potential (equivalent to +60 mV membrane potential). Extracellular solution with elevated K^+^ concentration was used to adjust the resting potential of the cell to 0 mV. Pipette solution contained 10 mM Ba^2+^ to silence endogenous K^+^ channels. Representative gating events of TRPV1_WT_ (**a**) and TRPV1_T406D_ (**b**) at −60 mV (i) induced by 0.3 μM capsaicin (ii) and by 0.3 μM capsaicin after 2 min incubation with 3.3 μM capsaicin (iii). Representative event distribution histograms showing the open and closed probability (*NP*_O/C_) of TRPV1 (**c**) and TRPV1_T406D_ (**d**). Statistical analysis of open and close probability revealed that the open probability of TRPV1_T406D_ was significantly reduced at −60 mV (i), and −60 mV + 0.3 μM capsaicin, whereas the pretreatment with 3.3 μM capsaicin induces similar N*P*_O_ of TRPV1_T406D_ as the TRPV1_WT_ (**e**). n = 3–10 independent recordings (unpaired *WR*-test, p < 0.05).

In order to study the functional effects of the TRPV1_T406D_ mutation at the level of single protein level, we analyzed the biophysical properties such as single-channel amplitude, open probability, and gating. To this end, we performed cell-attached recordings, equalized the membrane potential by using high [K^+^] extracellular solution and filled the patch pipettes with a solution containing 10 mM BaCl_2_ (solution E) to block endogenous K^+^ ion channels. Ion channel openings and closings were recorded for periods of >2 min. At least three independent single-channel measurements were conducted for every experimental setting (Fig. 8a,b). In general, single-channel amplitudes are dependent on both the electrochemical gradient and the ion channel pore characteristics. In both TRPV1_WT_ and TRPV1_T406D_ expressing CHO cells, we found single-channel amplitudes of 5–8 pA at −60 mV pipette potential (resulting in a membrane potential of +60 mV). Remarkably, the open state of the individual events did not show constant amplitudes, but tend to decrease from an initial high to a lower plateau conductance state. This behavior indicates a dynamic process within the pore, leading to alteration of conductance/permeability during gating. However, the mechanism behind this observation is not clear at the moment, but will be addressed in future studies. In order to analyze the open probability (*NP*_O_) of TRPV1_WT_ and TRPV1_T406D_, ion channels amplitude histograms were extracted from the data (Fig. 8c,d). Adding 0.3 μM capsaicin to the pipette solution increased the average open probability (*NP*_O_) of TRPV1_WT_ from 16.5 to 60.6% and the *NP*_O_ of TRPV1_T406D_ from 1.5 to 4.8%, demonstrating the low activity of TRPV1_T406D_ under these conditions (Fig. 8c,d). Pre-treating TRPV1_T406D_ expressing cells for two minutes with 3.3 μM capsaicin and adding 0.3 μM capsaicin to the pipette solution (after washout) led to priming of the receptor and increased the open probability to 88.8% (Fig. 8d), again reflecting the use-dependent activation of TRPV1_T406D_. The statistical analysis of the open and closed states of TRPV1_WT_ and TRPV1_T406D_ revealed that TRPV1_T406D_ spent significantly less time in the open state at +60 mV in the presence of 0.3 μM capsaicin, but priming TRPV1_T406D_ with 3.3 μM capsaicin shifted the open probability similar to TRPV1_WT_ (about 90%). These findings were in line with our previous whole-cell measurements, indicating that the use-dependent activation of TRPV1_T406D_ might be based on the engagement of different conformations.

In order to determine the state time constants of the open and closed states, the recorded single-channel events were further analyzed by calculating the open dwell-time distribution. Therefore the root square of the frequency versus the natural logarithm of time was plotted and fitted with polynomial functions to discriminate between the different open or closed states. Supplemental Fig. 7a,b shows representative dwell-time histograms for the activity of TRPV1_WT_ and TRPV1_T406D_. Similar to previous studies, we found three open states and four closed states^21^. Although the time constants of open states as well as the relative contribution of the states O1 to O3 seem to slightly vary with different experimental conditions (0.3 μM, with/without priming with 3.3 μM capsaicin), the open states were not significantly different between TRPV1_T406D_ mutant and TRPV1_WT_. The detailed analysis of the closed states C1 to C4 revealed an (compared with TRPV1_WT_) increased long time constant C4 in the TRPV1_T406D_ mutant, which is markedly reduced in both, time and relative contribution in the presence of 0.3 μM capsaicin after priming. However, closed states of TRPV1_WT_ are only slightly modulated by capsaicin (Supplemental Fig. 7c,d). The analysis of the gating properties point to the involvement of closed state modulation of TRPV1_T406D_ resulting in the observed use-dependent behavior. In summary, we have demonstrated in our mouse models that the oral pain responses mediated by TRPV1 receptors are modulated by Cdk5 activity and that co-expression of Cdk5, p35 and TRPV1 modulates ion channel desensitization *in vitro*. By mutating rTRPV1_T406_ (corresponds to T407 in mouse sequence) we were able to characterize TRPV1 ion channel properties in more detail. We investigated the functional parameters on the whole-cell (sensitivity, desensitization, voltage-dependence, activation kinetics), as well as on the single-channel level (open probability, open dwell-time distribution, and gating). Based on the altered TRPV1 ion channel function, we conclude that T406 (rat sequence) is crucial for TRPV1 receptor function and that the Cdk5-mediated phosphorylation of TRPV1 at position T406 has severe consequences on the transduction of potential painful stimuli.

## Discussion

Previous *in vivo* studies have demonstrated that genetically engineered Cdk5 mice have altered peripheral thermal^17^ and mechanical nociception^22^. The direct phosphorylation of transducers of noxious stimuli by Cdk5 may account for the alterations in nociception in these mice. Under this premise, the thermosensitive cation channel TRPV1 was identified to be a substrate for Cdk5 and inhibition of Cdk5 activity attenuates TRPV1-mediated Ca^2+^ influx in cultured DRG neurons, suggesting that Cdk5 modulates TRPV1 activity^17^. This hypothesis was tested *in vivo* using nociceptor specific Cdk5 conditional knockout mice, where abrogation of Cdk5-mediated phosphorylation of TRPV1 appeared to correlate with thermal hypoalgesia, as measured by increased paw and tail withdrawal latency^17^. In the current study, we have extended our *in vivo* analysis of Cdk5 activity and TPRV1-mediated pain responses by examining orofacial aversion to both the specific TRPV1 agonist capsaicin and to noxious heat (45 °C). Our results clearly demonstrate a correlation between Cdk5 activity and TRPV1-mediated pain transduction. Increased Cdk5 activity was correlated with a higher aversion to capsaicin and heat compared to wild type control (hyperalgesia), whereas the lack of Cdk5 activity showed less aversion to capsaicin (hypoalgesia). Thus, our findings indicate that Cdk5 phosphorylation of TRPV1 plays a crucial role in the mechanism of altered behavior and nociception in our Cdk5 mouse models.

In order to investigate the functional consequences of Cdk5-mediated TRPV1 phosphorylation on a molecular level, we used CHO cells co-expressing rTRPV1, Cdk5 and the neuron specific Cdk5 activator p35. We could show that in the presence of Cdk5 and p35, TRPV1-mediated currents induced by high concentrations of capsaicin (3.3 μM) did not desensitize, while the sensitivity to capsaicin was unaffected. As a functional consequence, a lack of TRPV1 desensitization most likely leads to a prolonged neuronal activity of sensory neurons, which then may cause hyperalgesia and/or allodynia. Similarly, Bhave *et al.* mutated potential PKA phosphorylation sites of the TRPV1 receptor to mimic or inhibit PKA-dependent phosphorylation. Replacing the target serine or threonine with aspartate or alanine, led to an altered receptor desensitization^23^. Also Numazaki and colleagues confirmed the involvement of two serine residues in protein kinase C-dependent TRPV1 potentiation by mutating S502 and S800 to alanine^24^. Similar mutagenesis studies were performed to identify the phosphorylation sites for CaMKII and c-Src kinase^14^,^25^. Pareek at al. were the first to describe a role of Cdk5 in phosphorylating TRPV1 leading to sensitization of its nociceptor function^17^. However, no data on the molecular impact of TRPV1 phosphorylation by Cdk5 were available so far. We generated several TRPV1_T406_ mutants aiming to mimic the phosphorylated or de-phosphorylated state of the receptor protein and to study the effect of amino acid residues owning different size and charge on the function of mutated TRPV1 ion channels. We could show that the introduction of both, negatively but also positively charged amino acid residues (as well as proline), reduced or even inhibited the Ca^2+^-dependent desensitization. Interestingly, TRPV1_T406D_ and TRPV1_T406E_ mutants exhibited a use-dependent behavior with low stimulus sensitivity and very slow kinetics at an initial activation, which was markedly enhanced and accelerated after priming with a high capsaicin concentration. In 2012, Xing *et al.* and in 2015, Liu *et al.* showed that Cdk5 positively regulates the TRPV1 membrane trafficking in nociceptors. They found that the Cdk5-dependent phosphorylation of the motor protein KIF13B promotes the TRPV1 trafficking process. Furthermore they showed that this regulatory mechanism contributes to inflammatory heat hyperalgesia^26^,^27^. To address the possible contribution of a stimulus-dependent receptor translocation to the observed change in activation kinetics, we investigated the membrane expression of GFP-tagged TRPV1_WT_ and TRPV1_T406_ mutants by means of TIRF microscopy, but could not observe increased GFP–fluorescence after priming the cells with a high concentration of capsaicin. These results suggest that the change in activation kinetics of TRPV1_T406D_ is not a consequence of increased membrane trafficking of receptors, but support our hypothesis that the accelerated activation kinetics is due to a use-dependent alteration in channel gating. Interestingly, TRPV1_T406D_ exhibits a use-dependent behavior and shows increased stimulus sensitivity (to capsaicin, low pH, and heat) as well as an enhanced voltage-dependence after priming with maximal capsaicin stimulation. Thus, our data suggest that the amino acid at position 406 significantly influences a basic function of the gating process. Our data demonstrate that a single amino acid in the intracellular N-terminus influences receptor properties and channel gating. Position 406 of the TRPV1 sequence is neither located within the capsaicin binding site or channel pore, nor directly related to the voltage sensor or another identified region of the protein responsible for heat or proton activation^28^. In order to shed light on the putative mechanism and molecular relations, we made use of the recently published high resolution (3–4 Å) structures of rTRPV1^18^,^19^. Three distinct rTRPV1 structures are available in the data bases: i) The apoproteine (PDB-3j5p), representing the closed state; ii) the capsaicin bound TRPV1 (PDB-3j5r), representing an intermediate state; and iii) the DkTx-RTX-bound TRPV1 structure (PDB-3j5q) representing the open state. Comparing these structures with focus on T406 revealed interesting details in the protein conformation. It appears that T406 is located in a flexible linker in close proximity to the TRP-domain, and the conformation of this linker seems to differ between the closed and open state (Supplementary Fig. 8). To scrutinize the putative role of T406 in the gating process of the pore, we made use of the all-atom simulation of TRPV1 by Zheng and Qin^29^, which is based on the high resolution structures of rTRPV1^18^,^19^. Based on their computations, the TRPV1 gating includes four functional parts. Listed in the motional order: i) ankyrin repeats domain (ARD) residue 110–357 → membrane proximal domain (MPD) residue 358–439 (contains T406) → C-terminal domain (CTD) 691–719 (including TRP-box) → Transmembrane domain (TMD) 5–6^29^. Furthermore, the authors analyzed the van der Waals (vdW) energy of the intracellular domains in the closed and open state. Within the intracellular domain, they found that several amino acids have higher vdW energy in the open state than in the closed state. According to their data, particularly T406, R409, and M412 show high vdW energy differences and are strategic positioned interfacing the MPD, the CTD and the TMD. The results from Zheng and Qin are in line with our data and interpretations and support the hypothesis that T406 participates in and modulates TRPV1 ion channel gating.

In summary, our results indicate that Cdk5-mediated phosphorylation of rTRPV1 at T406 plays an important role in the molecular process of transduction of nociceptive stimuli and pain signaling. Biophysical characterization of phosphorylated and non-phosphorylated but mutated TRPV1_T406_ receptors aimed to compare the effect of conformation and charge on receptor function. Our findings have considerably improved our understanding of the relationship between receptor structure and function, and we believe that this detailed knowledge will be the impetus for the development of new therapeutic approaches focused mainly on the modulation of receptor and channel properties rather than simply blocking channel function.

## Methods

### Generation of transgenic mice

p35 knockout (p35KO) and Cdk5 conditional knockout (Cdk5 CoKo) mice were maintained in C57BL6/129SVJ background and genotyped as described^17^,^30^. Cdk5 CoKo mice were generated by crossing Cdk5^f/−^ mice with SNS-Cre mice^17^. Transgenic p35 (Tgp35) mice were bred in FVBN background^31^. Age-matched wild-type mice served as controls. All animals were housed in standard cages in climate- and light-controlled rooms with free access to food and water. All experimental procedures were approved by the Animal Care and Use Committee of the National Institute of Dental and Craniofacial Research, National Institutes of Health and adhered to the guidelines of the IASP Committee for Research and Ethical Issue^32^.

### Mouse operant lickometer test

An operant lickometer test was used to test nociceptive responses to hot taste stimuli. Nociceptive sensitization was induced by 15 μM concentration of the TRPV1 agonist capsaicin (Sigma-Aldrich, St. Louis, MO, USA). Mice were deprived of water overnight (15 hrs), then placed in the lickometer cages (Habitest system, Coulbourn Instruments, USA). A computer-operated system monitored their licking events for 1 hour. Initially, the animals were tested with water (n = 5 sessions). Then, consumption/aversion to water with 15 μM capsaicin was monitored (n = 5 sessions). All mice were tested at the same time each day and then retested under the same conditions every other day.

### Mouse thermal operant behavioral assay

Assessment of thermal sensitivity was measured using an Orofacial Pain Assessment device (OPAD) (Stoelting)^33^,^34^. This device measures the changes in nociceptive behavior in trigeminal area after thermal stimulation. First, mice were trained to drink a sucrose (30%) reward while contacting two Pelletier-based thermodes (set to non-painful temperature 37 °C) to reach the reward. After completing 5 baseline training sessions, the animals were retested three times using heated thermodes to 45 °C. Mice were deprived of food and water overnight (for 12–15 hours) prior to testing to increase the incentive for sucrose acquisition. The Anymaze Software automatically tracked the number of licks or contacts with the thermodes and the time animals spent with the licking of the reward.

### Molecular biological methods

All cDNAs coding for the protein of interest were sub-cloned in pCDNA3 vectors. To identify co-transfected cells Cdk5 and p35 were C-terminally tagged with either mCherry (a red fluorescent protein) or CFP (cyan fluorescent protein). TRPV1_T406_ was replaced by 11 different native amino acids using overlap extension PCR or site-directed mutagenesis. Positively as well as negatively charged, aromatic, and non polar amino acids were inserted to mimic or inhibit the Cdk5-mediated phosphorylation. Additionally, proline-407 was also replaced by alanine. For TIRF microscopy, TRPV1_WT_, TRPV1_T406A_, and TRPV1_T406D_ were C-terminally tagged with GFP. For each residue, mutagenesis primers were designed including the desired mutation. Mutagenesis PCR was performed using PFU DNA polymerase (Agilent Technologies, Santa Clara, CA, USA) to prevent unwanted mutations. All mutants were confirmed and checked for mutations by DNA sequencing.

### Cell culture and transfection

Chinese hamster ovary (CHO) cells were cultured in MEM (PAN-Biotech, Aidenbach, Germany) and HEK293T (Human embryonic kidney) cells were grown in DMEM (Life Technologies, Darmstadt, Germany). Both media were supplemented with 10% (v/v) fetal calf serum and 1% (v/v) anti/anti (Sigma-Aldrich, Taufkirchen, Germany). Cells were cultured at 37 °C and 5% CO_2_ in Ø 10 cm cell culture dishes (TPP, Trasadingen, Switzerland). For patch-clamp experiments, 50–100 k cells were seeded on Ø 3 cm cell culture dishes (Sarstedt, Nümbrecht, Germany). For Ca^2+^-imaging measurements, 200 k cells were seeded on Ø 2.5 cm glass coverslips (Menzel, Braunschweig, Germany). After 6 to 32 hours, CHO or HEK293T cells were transfected with 4–6 μg of coding plasmid DNA. Transfection was performed via calcium phosphate precipitation method as described before^35^. 12–24 h after the transfection, CHO and HEK293T cells were used for electrophysiological and Ca^2+^ imaging experiments.

### Solutions and chemicals

In electrophysiological and Ca^2+^ imaging experiments following bath solutions were used (in mM): (A) NaCl 140, KCl 5, CaCl_2_ 2, MgCl_2_ 2, (B) NaCl 140, KCl 5, EGTA 5, MgCl_2_ 2, (C) KCl 140, MgCl_2_ 1, CaCl_2_ 0.1, EGTA 5. Utilized pipette solutions were (in mM): (D) CsCl 145, MgCl_2_ 2, CaCl_2_ 1, EGTA 11 and (E) CsCl 140, BaCl_2_ 10, MgCl_2_ 1, CaCl_2_ 0.1, EGTA 5. All solutions contained 10 mM HEPES and HCl were used to adjust the pH to 7.35. For low pH stimuli, solutions were adjusted to pH 6. If necessary, mannitol was added to adjust the osmolarity to 290 ± 10 mOsm for bath solutions and 300 ± 10 mOsm for pipette solutions. All chemical products were purchased at ROTH, Karlsruhe, Germany or Sigma-Aldrich. Capsaicin (Alomone, Jerusalem, Israel) was solved in DMSO to a final stock concentration of 33.3 mM to prepare various capsaicin dilutions (0.05–3.3 μM).

### Electrophysiological measurements

CHO cells were co-transfected with TRPV1 receptor variants and GFP, or with TRPV1, Cdk5-mCherry and p35-CFP. Patch-clamp experiments were performed with an inverse microscope with 40× objectives (Zeiss, Jena, Germany). A micro manipulator (Scientifica, Uckfield, UK) was used to place the patch pipette on transfected cells for whole-cell or cell-attached configuration. Patch pipettes were pulled from borosilicate glass (Science Products, Hofheim, Germany) by means of a horizontal pipette puller (Zeitz Instruments, Munich, Germany) and fire-polished to obtain a series resistance of 3–5 MΩ for whole-cell or 6–8 MΩ for cell-attached configuration. Capacitance and liquid junction potentials were adjusted using the built-in compensation algorithm of the amplifier. Between 60% and 90% of the series resistance was compensated. Patch-clamp recordings were performed at room temperature (22–24 °C) using a HEKA EPC10 amplifier (HEKA, Lambrecht, Germany) and HEKA Patchmaster software was used for data acquisition.

### Whole-cell recordings

In order to perform whole-cell recordings, transfected CHO cells were clamped at their assumed resting potential of −60 mV. Voltage ramp protocols were applied continuously every second, beginning with −60 mV for 100 ms, followed by a voltage step to −100 mV for 100 ms in order to record inward directed currents. A 500 ms long linear ramp segment from −100 mV to +100 mV was applied and held for 100 ms in order to conduct outward directed currents. The voltage ramp protocol was finished by a last step back to −60 mV. Voltage step protocols begun with an initial constant segment at 0 mV for 20 ms, followed by steps from −120 mV to +160 mV in 20 mV increments ending with a step to +60 mV for 20 ms. Data of whole-cell voltage ramp and voltage step measurements were collected with a sample rate of 2 kHz or 50 kHz, respectively, and low pass-filtered at 2.9 kHz. To induce TRPV1-mediated currents, an air pressure driven 8 in 1 application system (ALA Scientific Instruments, Farmingdale, NY, USA) was used to apply different stimuli, such as capsaicin or low pH, directly onto the recorded cell.

### Cell-attached single channel recordings

Single TRPV1 channels were recorded in cell-attached configuration. Only patches with a series resistance >1 GΩ and leak currents <50 pA were recorded. To equilibrate the membrane potential to zero the bath solution contained 140 mM K^+^ instead of Na^+^ (solution C). Additionally to Cs^+^, the pipette solution contained Ba^2+^ in order to block endogenous K^+^ ion channels (solution E). Depending on the experimental approach, 0.3 μM capsaicin was added to the pipette solution. In a second approach, cells were pretreated with 3.3 μM capsaicin in the bath solution for two minutes prior to the measurement. Pipette potential was clamped at −60 mV and data were collected for >2 min with a sampling rate of 10 kHz filtered with a 2.9 kHz Bessel filter. To illustrate single channel recordings, a 1 kHz low-pass filter was added.

### TIRF microscopy

For the TIRF microscopy experiments CHO cells were seeded on glass cover slips and were transiently transfected with the respective TRPV1-GFP plasmids. The GFP fluorescence (488 nm) of the transfected cells was monitored 24 h later in a Leica AF 6000LX system using a HCX PL APO 100×/1.47 oil objective. To gain the best signal to noise ratio the penetration depth was set to 90 nm. GFP fluorescence was imaged in Ca^2+^-containing Ringer’s solution and 5 min after the application of capsaicin, which leads to a final capsaicin concentration of 6.6 μM. The selected cells were used to analyze the time course of the fluorescence by capturing images every 5 s.

### Fura-2 Ca^2+^ imaging

Ca^2+^ imaging experiments were performed using a ZEISS live cell imaging setup based on an Observer Z.1 (Zeiss, Jena, Germany) and images were recorded by using 40× and 20× objective lens as described before^36^. Temperature and capsaicin stimuli were applied via an 8 in 1 inline solution heater (Warner Instruments, Hamden, USA). 30 min before the measurement, cells were loaded with 2 μM Fura-2/AM in cell culture medium at 37 °C. The cell culture medium was replaced by Ringer’s solution (solution A). Illumination control and image recording were performed using a Lambda DG4 high-speed wavelength switcher (Sutter instruments, Novato, USA) and the Zen imaging software (Zeiss, Jena, Germany). Ca^2+^ signals were expressed as ratio of the fluorescence intensity during excitation with 340 nm or 380 nm (*F*_*340*_/*F*_*380*_).

### Statistical analysis

All data are expressed as mean ± SEM. The statistical evaluation of mouse behavioral tests was done with GraphPad Prism software, version 6 (GraphPad, San Diego, CA, USA). Statistical differences between the WT and p35 overexpressing mice (FVBN background) were assessed by unpaired t-test. Statistical differences between the WT and p35 downregulated mice (C57BL6 background) were assessed by One-way ANOVA followed by Dunnett’s multiple comparisons test. Data of electrophysiological and Ca^2+^ imaging experiments are presented as mean ± SEM (*n* = number of cells). Data were visualized and analyzed using Igor Professional 6.37 (Wavemetrics, Portland, USA), TAC ×4.3.3 and TACfit (Bruxton Corporation, Seattle, USA), Microsoft Office (Microsoft Corporation, Redmond, USA) and CorelDraw X6 (Corel Corporation, Ottawa, Canada). Data were tested for normal distribution prior to the statistical analysis. Parametric data were tested with Student’s *t*-test and nonparametric data with the Wilcoxon signed-rank test. Level of significance was set at p < 0.05. The analysis of the capsaicin concentration/response-relationship and of the conductance/voltage-relationship, in order to calculate EC_50_ or V_1/2_, was performed by the Wavemetrics Igor Professional 6.37 software.

### Hill’s Equation (Equation 1)


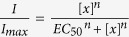
; I = current, I_max_ = maximal current at saturating concentration, x = concentration of tested agonist, EC_50_ = the calculated concentration that elicits 50% of maximal current, and n = Hill coefficient.

### Sigmoidal function (Equation 2)


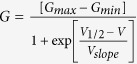
; G = conductance, G_max_ = maximal conductance, G_min_ = minimal conductance, V = applied voltage, V_1/2_ = voltage at half maximal conductance and V_slope_ = slope of the activation curve.

## Additional Information

**How to cite this article**: Jendryke, T. *et al.* TRPV1 function is modulated by Cdk5-mediated phosphorylation: insights into the molecular mechanism of nociception. *Sci. Rep.* **6**, 22007; doi: 10.1038/srep22007 (2016).

## Supplementary Material

Supplementary Information
