# Supplementary material for: TRPV1 function is modulated by Cdk5-mediated phosphorylation: insights into the molecular mechanism of nociception
Source: Sci Rep. 2016 Feb 23;6:22007. doi: 10.1038/srep22007 (PMC4763283; doi:10.1038/srep22007)
Supplement: Supplementary Information [file srep22007-s1.doc]

**TRPV1 function is modulated by Cdk5-mediated phosphorylation: insights into the molecular mechanism of nociception**

**Thomas Jendryke^1^, Michaela Prochazkova^2^, Bradford Hall^2^, Grégory C. Nordmann^1^, Moritz Schladt^1^, Vladimir M. Milenkovic^1^, Ashok B. Kulkarni^2^ and Christian H. Wetzel^1*^**

**Supplementary information**


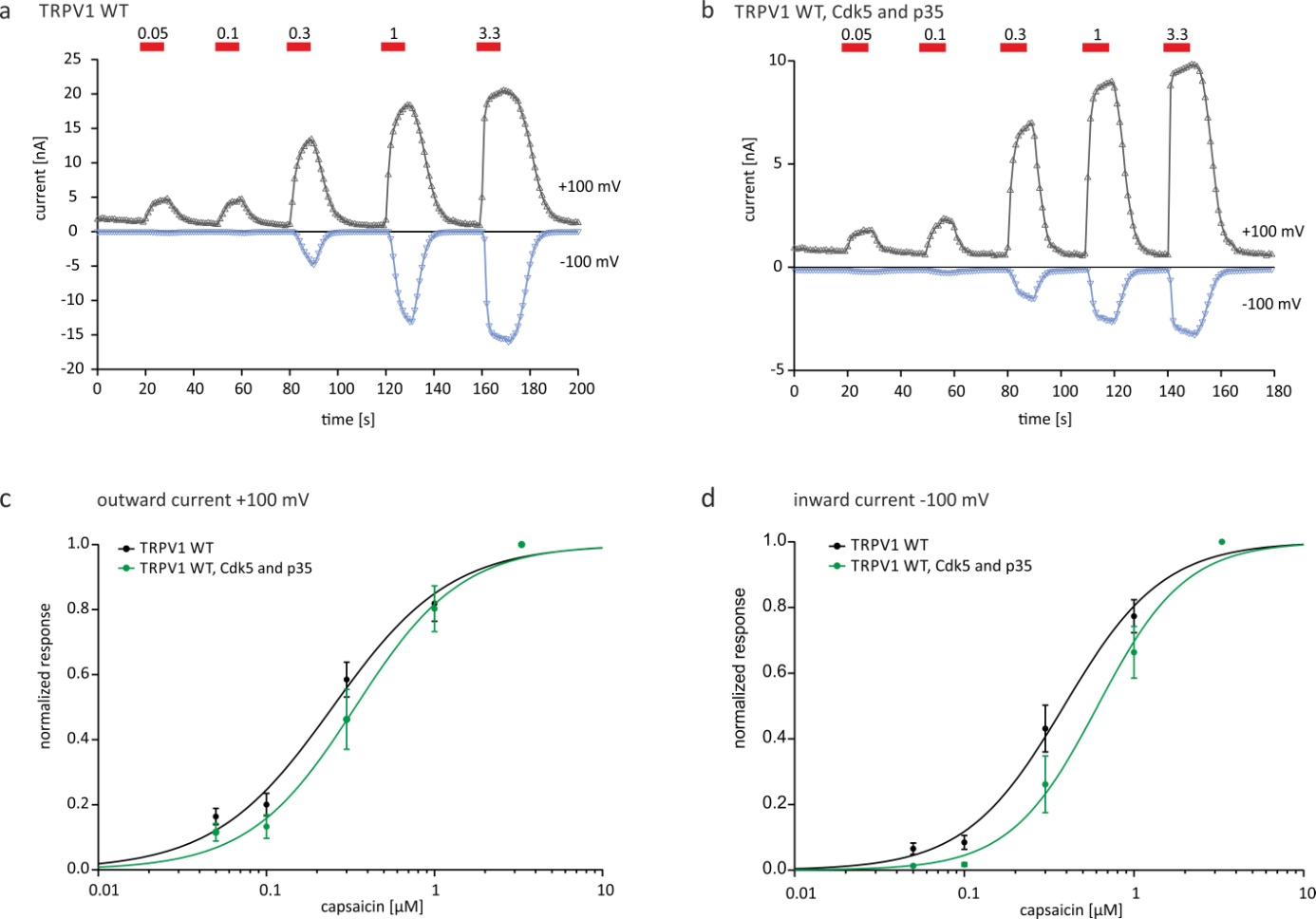


**Supplemental Figure 1.** Capsaicin concentration-response relationship of CHO cells co-expressing TRPV1 or TRPV1, Cdk5, and p35. TRPV1-mediated outward and inward currents were recorded by voltage-ramp protocols (‑100 mV to +100 mV) in absence of extracellular Ca^2+^. Representative recordings of TRPV1 (**a**) or TRPV1, Cdk5 and p35 (**b**) mediated currents. For analysis the data were normalized to the maximal current. Apparent affinity (EC_50_) was calculated by means of the Hill equation. The EC_50_ of the outward (**c**) and inward (**d**) currents were not significantly different between TRPV1 and TRPV1, Cdk5, and p35 expressing cells. EC_50_ values: TRPV1 outward: 0.25 ± 0.05 µM, inward: 0.63 ± 0.13 µM; TRPV1, Cdk5, and p35 outward: 0.28 ± 0.04 µM, inward: 0.55 ±0.08 µM; unpaired *t-test* +100 mV: p>0.05, -100 mV: p>0.05; n=10-18


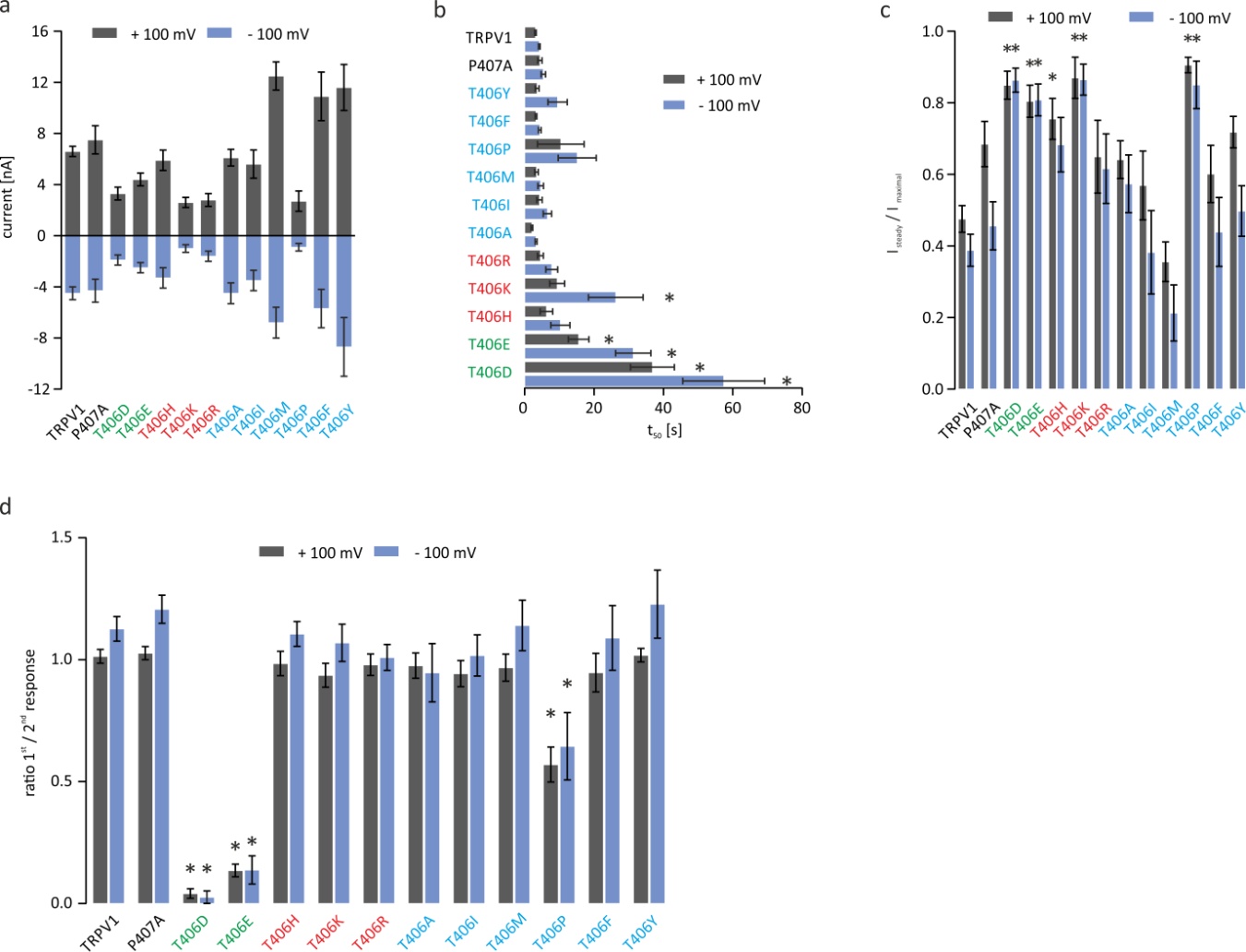


**Supplemental Figure 2.** Functional properties of various TRPV1_T406_ mutants. Maximum TRPV1-mediated currents in transiently transfected CHO cells induced by 3.3 μM capsaicin (200 s) in the presence of extracellular Ca^2+^ (**a**). Activation kinetics is represented as time to half-maximal activation t_50_ (**b**) and desensitization is represented as ratio I_steady_/I_maximal_ (**c**). Sensitization of TRPV1_T406_ mutants represented as ratio of 1^st^/2^nd^ response to pH 6, in the absence of extracelluar Ca^2+^ (**d**). Data are represented as mean ± SEM values of n=5-22 independent experiments.


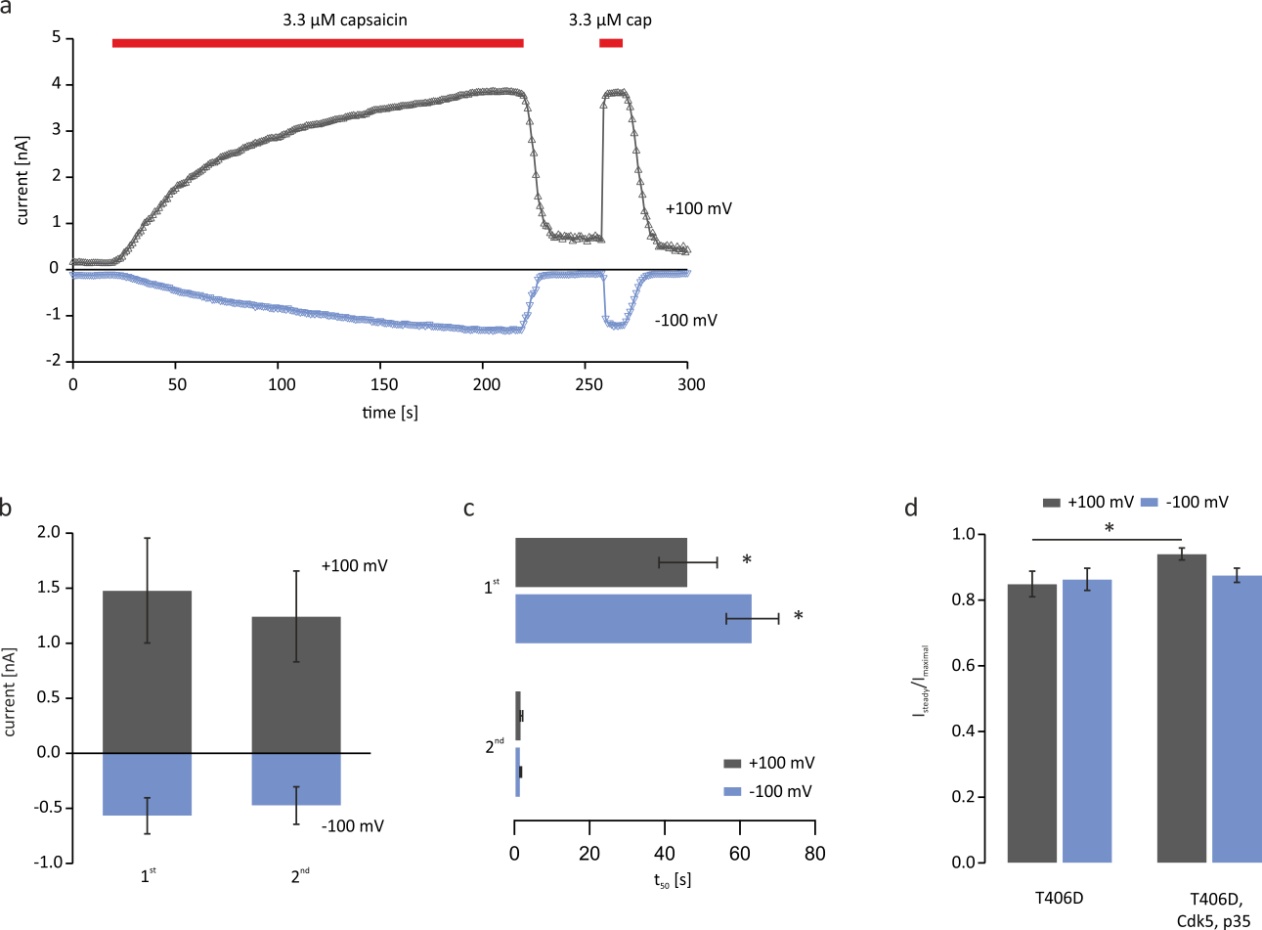


**Supplemental Figure 3.** TRPV1-mediated currents of cells co-expressing TRPV1_T406D_, Cdk5, and p35. Representative TRPV1_T406D_-mediated inward (-100 mV) and outward (+100 mV) currents, induced by application of 3.3 µM capsaicin in the presence of Ca^2+^-containing Ringer’s solution (**a**). Maximal amplitude (**b**), time to half maximal activation (t_50_) (**c**), and statistical analysis of the desensitization, represented as ratio (I_steady_/I_maximal_) (**d**). Mean ± SEM of n=6 independent measurements, asterisk (*) indicates significant differences (paired or unpaired *WR*-test, respectively; p<0.05).


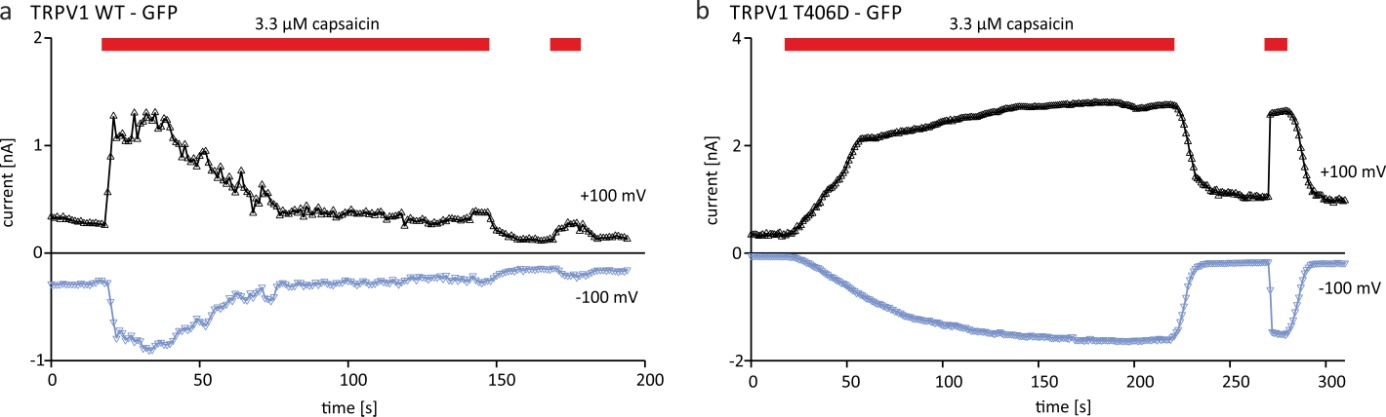


**Supplemental Figure 4.** TRPV1-mediated currents of TRPV1-GFP fusion proteins. Representative TRPV1-mediated currents of TRPV1_WT_-GFP (**a**), or TRPV1_T406D_-GFP (**b**), induced by the application of 3.3 µM capsaicin. Inward (‑100 mV) and outward (+100 mV) currents were recorded by means of voltage-ramp protocols in the presence of Ca^2+^-containing Ringer’s solution. n=3-5 independent recordings.


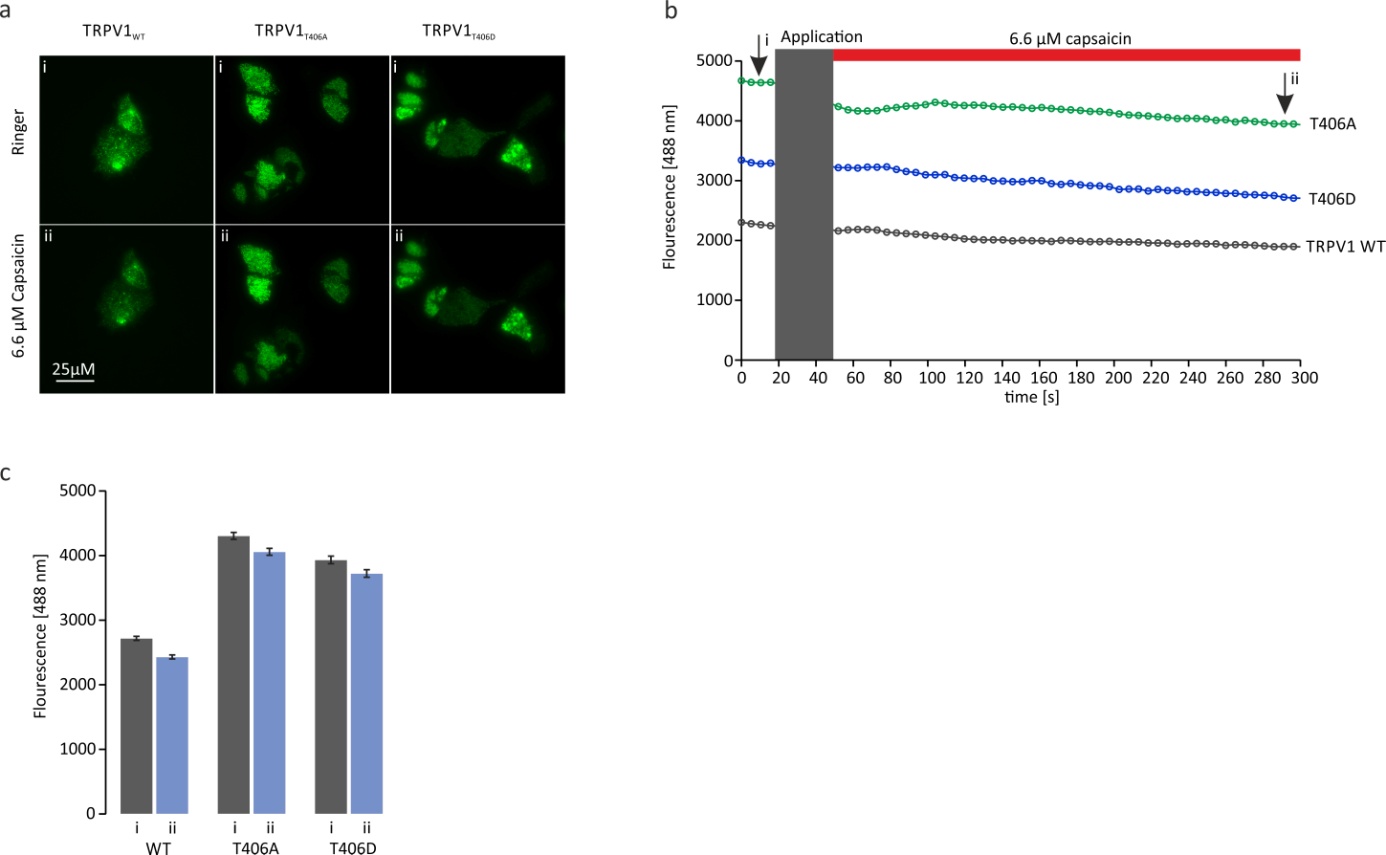


**Supplemental Figure 5.** Analysis of plasma membrane expression of GFP-tagged TRPV1 receptors in CHO cells. Representative GFP fluorescence images of CHO cells expressing TRPV1_WT_-GFP, TRPV1_T406A_-GFP or TRPV1_T406D_-GFP fusion proteins in Ringer’s solution and after 5 min incubation with 6.6 µM capsaicin (**a**). Time course of the GFP fluorescence; image capturing was stopped during the capsaicin application (grey bar). Representative fluorescence intensities of selected regions of interest (ROI) of TRPV1_WT_-GFP, TRPV1_T406A_-GFP or TRPV1_T406D_-GFP expressing cells (**b**). Mean GFP fluorescence in Ringer’s solution (i) and 5 min after the application of capsaicin (ii) (**c**). Mean values of n=31-59 recordings.


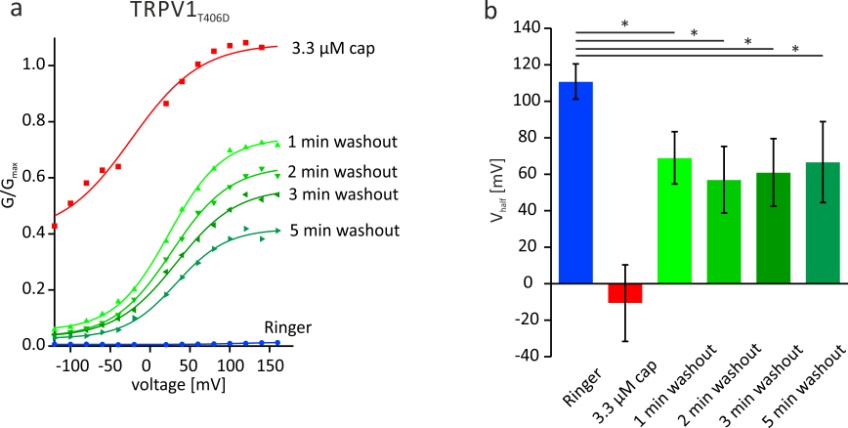


**Supplemental Figure 6.** Conductance/voltage analysis of TRPV1_T406D_. The normalized conductance (G/G_max_) was fitted by means of a sigmoidal function in order to analyze the V_1/2_. Representative conductance/voltage relationship of TRPV1_T406D_ under Ringer’s solution, 3.3 µM capsaicin and 1, 2, 3, and 5 min after the capsaicin washout (**a**). Statistical analysis revealed a significant decrease of V_1/2_ after the washout of capsaicin (paired *t-test* p<0.05; n=4) (**b**).


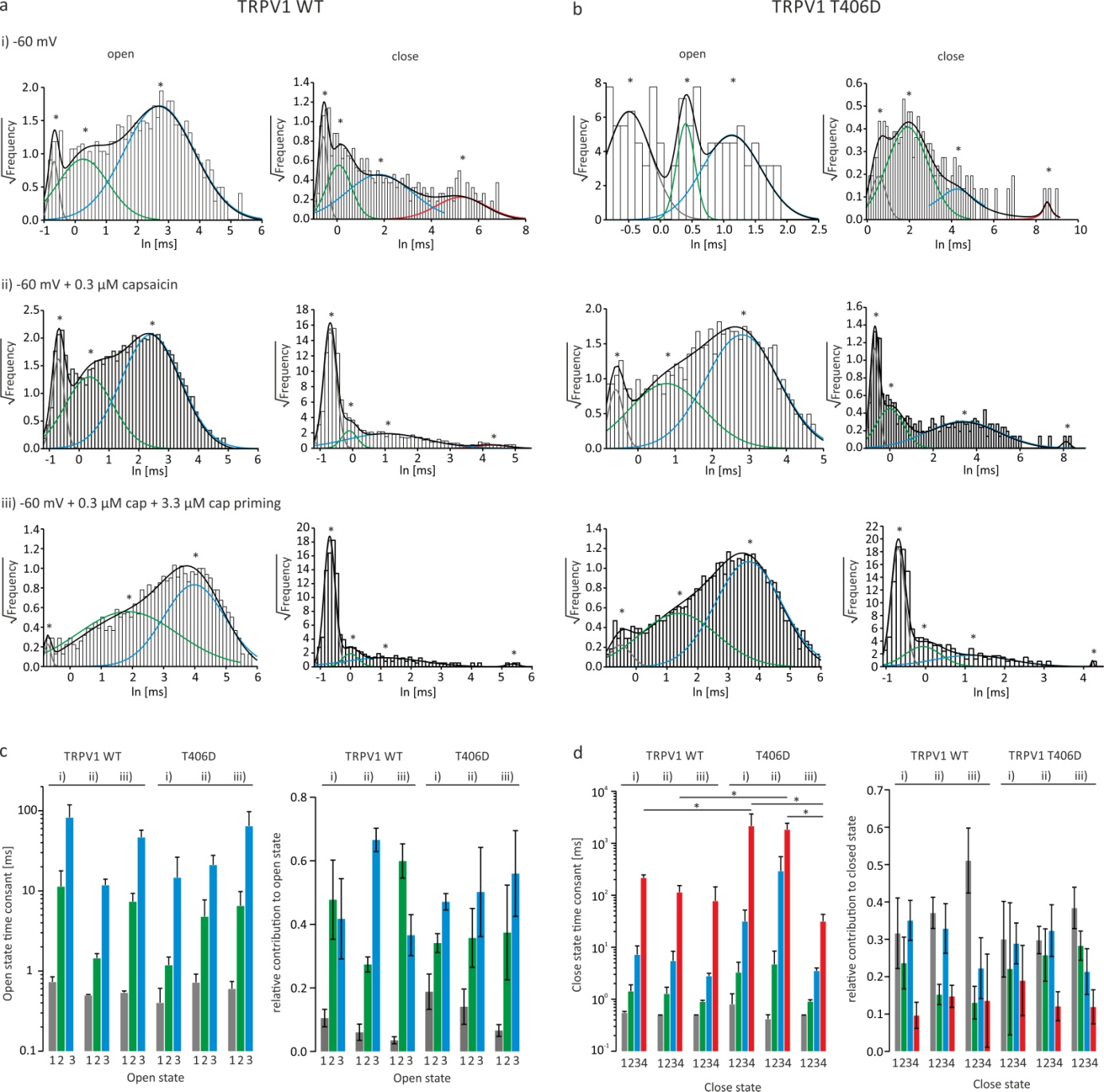


**Supplemental Figure 7.** Dwell-time analysis of TRPV1_WT_ and TRPV1_T406D_ single-channel recordings. Representative open and closed dwell-time histograms of TRPV1_WT_ (**a**) and TRPV1_T406D_ (**b**) at ‑60 mV (i), ‑60 mV + 0.3 µM capsaicin (ii), and ‑60 mV + 0.3 µM capsaicin after priming with 3.3 µM capsaicin (iii). The polynomial fit of TRPV1_WT_ and TRPV1_T406D_ dwell-time distributions revealed 3 open states (O1, O2, and O3) as well as 4 closed states (C1,C2,C3, and C4), marked as *. The open state time constants and their relative contribution revealed no significant differences between TRPV1_WT_ and TRPV1_T406D_ (**c**). However, the closed state C4 was significantly (*) higher under Ringer’s condition, compared to the corresponding TRPV1_WT_ C4 and the TRPV1_T406D_ C4 after priming with 3.3 µM capsaicin (unpaired *WR*-test p<0.05; n=3-10) (**d**).


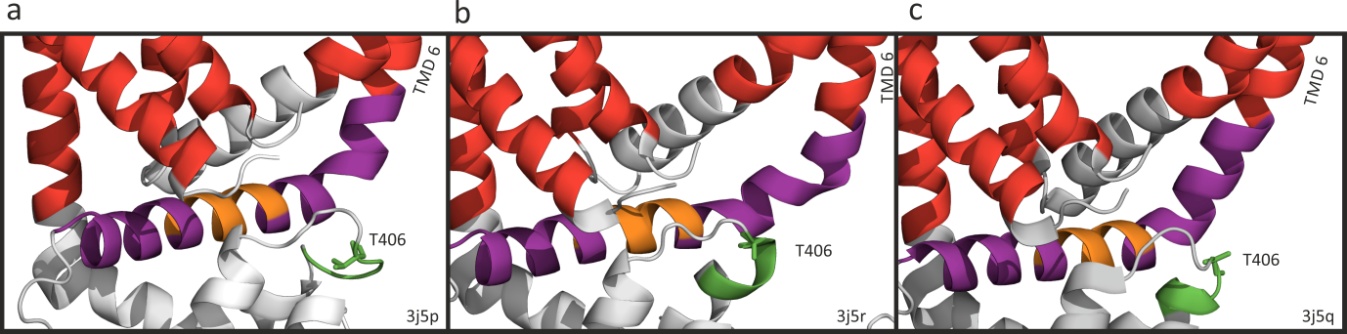


**Supplemental Figure 8.** TRPV1 structure in distinct conformations revealing structural changes in the vicinity of position T406. Apo-protein (**a**), capsaicin bound (**b**) and RTX/DkTx bound (**c**) reveal that T406 is located in a flexible linker close to the TRP domain including the TRP box. Relevant TRPV1 structures were color coded: **Red** transmembrane domain (TMD) 1-6, **Purple** TRP domain, **Orange** TRP box, and **Green** Cdk5 consensus site.


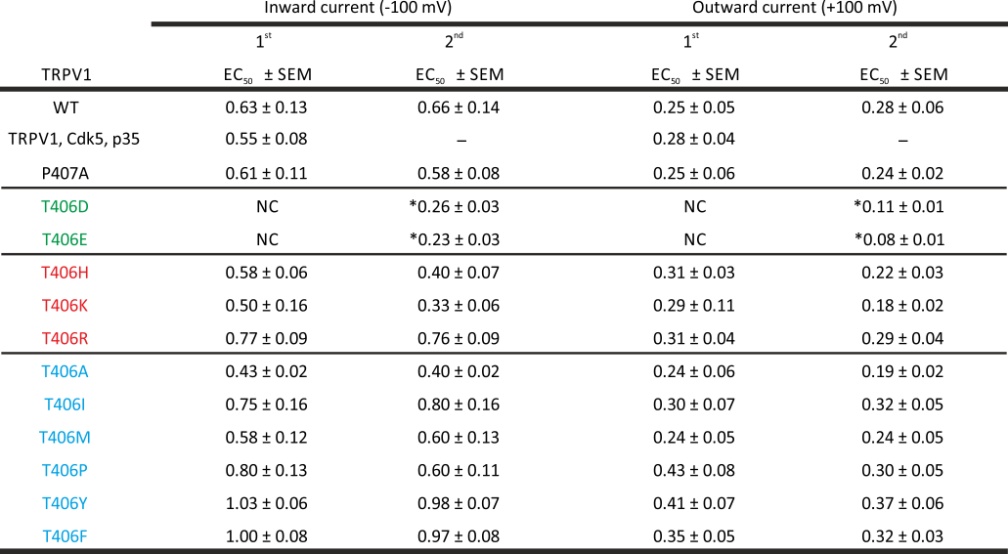


**Supplemental Table 1.** EC_50_ values of TRPV1 receptor variants calculated by concentration/response-relationship measurements of inward and outward currents (under Ca^2+^-free conditions to prevent desensitization).
